# Cardiac MRF using rosette trajectories for simultaneous myocardial T_1_, T_2_, and proton density fat fraction mapping

**DOI:** 10.3389/fcvm.2022.977603

**Published:** 2022-09-20

**Authors:** Yuchi Liu, Jesse Hamilton, Yun Jiang, Nicole Seiberlich

**Affiliations:** ^1^Department of Radiology, University of Michigan, Ann Arbor, MI, United States; ^2^Department of Biomedical Engineering, University of Michigan, Ann Arbor, MI, United States

**Keywords:** cardiac MRF, T_1_ mapping, T_2_ mapping, PDFF, rosette trajectory

## Abstract

The goal of this work is to extend prior work on cardiac MR Fingerprinting (cMRF) using rosette k-space trajectories to enable simultaneous T_1_, T_2_, and proton density fat fraction (PDFF) mapping in the heart. A rosette trajectory designed for water-fat separation at 1.5T was used in a 2D ECG-triggered 15-heartbeat cMRF sequence. Water and fat specific T_1_ and T_2_ maps were generated from the cMRF data. A PDFF map was also retrieved using Hierarchical IDEAL by segmenting the rosette cMRF data into multiple echoes. The accuracy of rosette cMRF in T_1_, T_2_, and PDFF quantification was validated in the ISMRM/NIST phantom and an in-house built fat fraction phantom, respectively. The proposed method was also applied for myocardial tissue mapping of healthy subjects and cardiac patients at 1.5T. T_1_, T_2_, and PDFF values measured using rosette cMRF in the ISMRM/NIST phantom and the fat fraction phantom agreed well with the reference values. In 16 healthy subjects, rosette cMRF yielded T_1_ values which were 80~90 ms higher than spiral cMRF and MOLLI. T_2_ values obtained using rosette cMRF were ~3 ms higher than spiral cMRF and ~5 ms lower than conventional T_2_-prep bSSFP method. Rosette cMRF was also able to detect abnormal T_1_ and T_2_ values in cardiomyopathy patients and may provide more accurate maps due to effective fat suppression. In conclusion, this study shows that rosette cMRF has the potential for efficient cardiac tissue characterization through simultaneous quantification of myocardial T_1_, T_2_, and PDFF.

## Introduction

Quantitative cardiac MRI is a powerful tool which can enable comprehensive tissue characterization for cardiac disease diagnosis. In particular, T_1_ and T_2_ mapping in the heart have been shown to be more sensitive to pathological changes than traditional T_1_- and T_2_-weighted images, including in cases of myocardial inflammation, fibrosis, myocarditis, infarcts, and edema, etc., (1–3). In addition, elimination of fat signals can reduce errors in these quantitative maps caused by water-fat partial volume effects, and quantitative proton density fat fraction (PDFF) mapping may provide additional value in diagnosing diseases like intramyocardial fat infiltration (4, 5). Recently, studies have shown that epicardial adipose tissue may play a role in COVID-19 myocardial inflammation, and quantification of epicardial fat volume may potentially aid evaluating this risk factor for COVID-19 complications (6).

When collected as part of the clinical routine, T_1_ and T_2_ mapping and fat imaging in the myocardium are often performed in separate scans and thus require long scan times with multiple breath holds. Multi-parametric mapping methods such as cardiac Magnetic Resonance Fingerprinting (cMRF) (7) are potentially more efficient because they can provide multiple quantitative measurements in a single scan. Previously, the Dixon method has been incorporated in the cMRF framework using multi-echo radial acquisitions to enable T_1_, T_2_, and PDFF quantification (8). Alternatively, rosette trajectories have also been used in the cMRF sequence to achieve water-fat separation along with myocardial T_1_ and T_2_ mapping (9). Rosette trajectories can be designed to sample the center of k-space multiple times during one readout, resulting in the suppression of signals at certain off-resonance frequencies due to dephasing. In other words, rosette trajectories can be used to generate a “pass band” and “null band” in the spectral dimension. This feature has been used for water-fat separation (10), chemical shift encoding (11, 12), and simultaneous multi-slice imaging (13). While the previous rosette cMRF work achieved water-fat separation, quantification of fat fraction was found unreliable due to the nature of the proton density estimates generated by pattern matching (9). The goal of this work is to extend rosette cMRF to enable quantitative PDFF measurements using Hierarchical IDEAL along with myocardial T_1_ and T_2_ mapping from a single scan.

## Materials and methods

### Pulse sequence design

**Figure 1:**
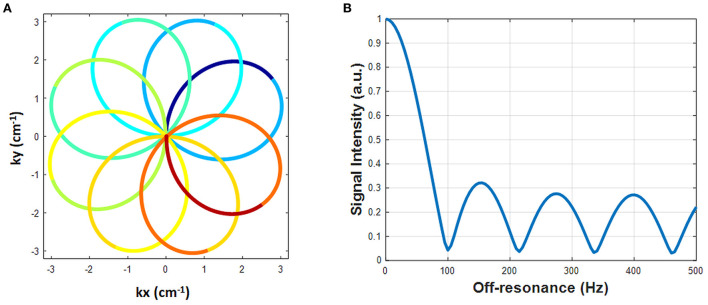
The designed rosette trajectory and its spectral response. **(A)** The rosette trajectory used in this work, where the segments used to generate images at different echo times are indicated by different colors. **(B)** Spectral response of the trajectory. Fat signals at −220 Hz are suppressed to 5.3%.

A rosette trajectory with eight lobes and a readout duration of 7.7 ms (Figure 1A) was designed to suppress signals at −220 Hz (the main resonance frequency of fat at 1.5T). The time optimal gradient design software package developed by Vaziri and Lustig (14, 15) was used for the gradient waveform design according to the following criteria: maximum gradient amplitude 23 mT/m, maximum slew rate 145 T/m/s, FOV 300 × 300 mm^2^, matrix size 192 × 192, in-plane resolution 1.56 × 1.56 mm^2^. Simulation studies show that this trajectory suppresses 94.7% of the signal at−220 Hz (Figure 1B). This readout trajectory was incorporated into a previously reported 15-heartbeat ECG-triggered cMRF sequence structure (9) with flip angles ranging from 4 to 25 degrees. A constant TR of 9.7 ms and TE of 1.39 ms were used. A total of 26 repetitions of this acquisition were collected at late diastole during each heartbeat, resulting in an acquisition window of ~250 ms per heartbeat and a total of 390 highly undersampled images (one image per TR) over 15 heartbeats. The rosette trajectory was rotated by the golden angle (111°) between TRs. A slice thickness of 8 mm was employed in all phantom and *in vivo* experiments. All data were acquired at the resonance frequency of water.

### Dictionary generation and image reconstruction

An individual dictionary was simulated for each subject that models the subject's cardiac rhythm (7) and includes corrections for slice profile and preparation pulse efficiency (16). The dictionary resolution, denoted by min:step:max, was (10:10:2000, 2050:50:3000 3200:200:4000 4500 5000) ms for T_1_ in the heart; (10:10:90, 100:20:1000, 1040:40:2000, 2050:50:3000) ms for T_1_ in phantoms; (4:2:80, 85:5:120, 130:10:300, 350:50:500) ms for T_2_ in the heart; (2:2:8, 10:5:100, 110:10:300, 350:50:1100) ms for T_2_ in phantoms. The dictionary was compressed along the time dimension using singular value decomposition (SVD) (17). A threshold was set to preserve 99.9% of the signal energy, resulting in the first six singular values retained.

The cMRF k-space data were first compressed along the coil dimension using SVD to preserve 98% of the signal energy. Then the k-space data were projected to the subspace derived from the SVD of the dictionary as described above, resulting in six “coefficient images” by applying the NUFFT (18). These six coefficient images correspond to the six largest singular values and aliasing artifacts are greatly reduced in them. When rosette data are collected at the resonance frequency of water (as described above), water signal is preserved, but signal from fat is suppressed, resulting in images which depict water but not fat. As in previous work (9), fat images were generated by demodulating the acquired data at the resonance frequency of fat with a single-peak fat model and then reapplying the projection and NUFFT to the k-space data.

Additionally, a B_0_ map was computed by generating two images with different echo times by gridding the first and second halves of the readout (lobes 1–4 for echo 1 and lobes 5–8 for echo 2) and calculating the phase difference of these two images (9). B_0_ correction was performed on both the water and fat coefficient images by demodulating the k-space data at a series of frequencies from−150 Hz to 150 Hz with a step size of 5 Hz. The final B_0_ corrected image combines pixels demodulated at the true resonance frequency according to the B_0_ map. The two sets of B_0_ corrected coefficient images, one for water and one for fat, were then matched to the compressed dictionary using direct pattern match to generate the final T_1_ and T_2_ maps, and proton density images for water and fat, respectively. For spiral cMRF data, similar dictionary generation and pattern matching processes were performed without B_0_ correction due to the lack of a co-registered B_0_ map.

To generate quantitative PDFF maps, the data were processed as multi-echo acquisitions using Hierarchical IDEAL (19) in a separate process from MRF reconstruction (pattern matching was not involved). The 8-lobe trajectory was divided into nine segments (Figure 1A). The first and the last segment were half lobes going from the center to the edge of k-space and rewinding from the edge of k-space back to the center, respectively. The other seven segments started and ended at the edge of k-space with a zero-crossing in the middle. Because the images generated from single segments were highly undersampled, an SVD was performed along the time dimension to reduce aliasing artifacts. Data from each segment were projected onto a low-dimensional subspace of rank six derived from the SVD of the dictionary as described above. Subspace images corresponding to the first singular value from each of the nine rosette segments served as multi-echo images. TE of each echo was defined as the time of the zero-crossing of each segment; the TEs of the nine echoes were: 1.39, 2.46, 3.4, 4.34, 5.28, 6.22, 7.16, 8.1, and 9.2 ms. These multi-echo images and their corresponding TEs served as the inputs to the Hierarchical IDEAL toolbox. Note that B_0_ correction was not performed on these multi-echo images prior to the IDEAL processing because B_0_ fitting was already embedded in the IDEAL algorithm. A six-peak fat model was used in the Hierarchical IDEAL algorithm and outputs of the toolbox were a water image and a fat image. A PDFF map was calculated from the water and fat images using a noise correction method (20) to reduce bias in the regions where either water or fat image has low SNR according to the following equation:


(1) $$PDFF=\{\begin{array}{c} \frac{\left|M_{0}^{fat}\right|}{\left|M_{0}^{fat}+\;M_{0}^{water}\right|}\;if\;\left|M_{0}^{fat}\right|>\left|M_{0}^{water}\right|\\ 1-\frac{\left|M_{0}^{water}\right|}{\left|M_{0}^{fat}+\;M_{0}^{water}\right|}if\;\left|M_{0}^{water}\right|>\left|M_{0}^{fat}\right| \end{array}$$

where $M_{0}^{water}$ and $M_{0}^{fat}$ are pixel-wise signal intensities of the water and fat images generated from Hierarchical IDEAL, respectively.

### Phantom experiments

All experiments were performed on a 1.5T scanner (Siemens Sola, Erlangen, Germany). Rosette cMRF data were collected in the T_2_ layer of the ISMRM/NIST MRI system phantom (21, 22) to validate the accuracy of water T_1_ and T_2_ quantification. The mean and standard deviation of the T_1_ and T_2_ values within a physiological range obtained using rosette cMRF were compared with gold standard values measured using inversion recovery and single echo spin echo methods.

The accuracy of rosette cMRF in PDFF quantification was validated using an in-house developed fat fraction phantom (23). This phantom had one vial filled with peanut oil, one vial filled with water solution, and the rest of the five vials filled with a mixture of peanut oil and water solution to target a range of PDFF values from 10% to 50%. The water solution contained 43 mM sodium dodecyl sulfate, 43 mM sodium chloride, 3.75 mM sodium azide, and 0.3 mM gadolinium. For all vials except for the one with pure peanut oil, agar (2% w/v) was added over heat and the vials formed a solid gel after cooling to room temperature. Note that super-paramagnetic iron oxide was not added in this phantom compared to the original recipe in (23). Considering imperfect operations which might cause water solution and/or peanut oil losses in transfer, the actual PDFF values were measured using a three-point GRE sequence with optimal echo times at 1.5T (1.9/3.4/4.9 ms). The three-point GRE data were processed using the Hierarchical IDEAL toolbox in the same way as for rosette cMRF 9-echo data, and the results were used as the gold-standard PDFF values.

Both phantoms were scanned in an axial orientation using a 20-channel head coil with simulated ECG signals at 60 bpm. For both phantoms, ROIs in each vial were drawn manually. The mean and standard deviation in T_1_ and T_2_ values in the ISMRM/NIST phantom and PDFF values in the fat fraction phantom for each ROI were compared to reference values using a linear regression test.

### *In vivo* experiments

Sixteen healthy subjects and two patients with suspected cardiomyopathy were scanned after written informed consent in this IRB-approved study. Mid-ventricular level short axis slices in the heart were acquired using the proposed rosette cMRF sequence and the original spiral 15-heartbeat cMRF sequence with the same flip angle pattern and acquisition window (9). Conventional T_1_ and T_2_ maps (MOLLI and T_2_-prepared bSSFP) were also collected in twelve of the healthy subjects and patients. The conventional scans are part of the Siemens MyoMaps product and used the following parameters: FOV 300 × 300 mm^2^, matrix size 192 × 192, GRAPPA R = 2 and 6/8 Partial Fourier acquisition. The 5(3)3 version of MOLLI was used with an acquisition window of 285.2 ms. The conventional T_2_ mapping scan used a 1(3)1(3)1 acquisition scheme with T_2_ preparation times of 0, 25, 55 ms and an acquisition window of 242 ms. Shimming was performed over the volume of the heart instead of the entire FOV to achieve better B_0_ field homogeneity. For patient scans, rosette cMRF, spiral cMRF and MOLLI were also performed ~10 min after contrast agent injection.

ROIs over the myocardial wall were drawn manually in segments 7–12 of the standardized AHA model. The mean and standard deviation in T_1_ and T_2_ values of each ROI as well as over the entire myocardium were calculated. In healthy subjects, a student's *t*-test was used to compare T_1_ and T_2_ measurements using rosette cMRF, spiral cMRF, and conventional T_1_/T_2_ mapping sequences. Significant difference was considered with *P* < 0.05.

To further investigate the effects of fat suppression on T_1_ and T_2_ measurements using rosette cMRF *in vivo*, a water-fat “unseparated” situation was mimicked by combining the water and fat information from the rosette trajectory. To this end, using rosette cMRF data in all healthy subjects, the k-space data demodulated at the fat frequency (fat signals with water suppression) were added to the original acquired k-space data (water signals with fat suppression). Then image reconstruction and pattern matching were performed in the same way as for spiral cMRF data. The mean and standard deviation in T_1_ and T_2_ values of the ROIs described above were calculated and compared with rosette and spiral cMRF measurements.

## Results

### Phantom data

**Figure 2:**
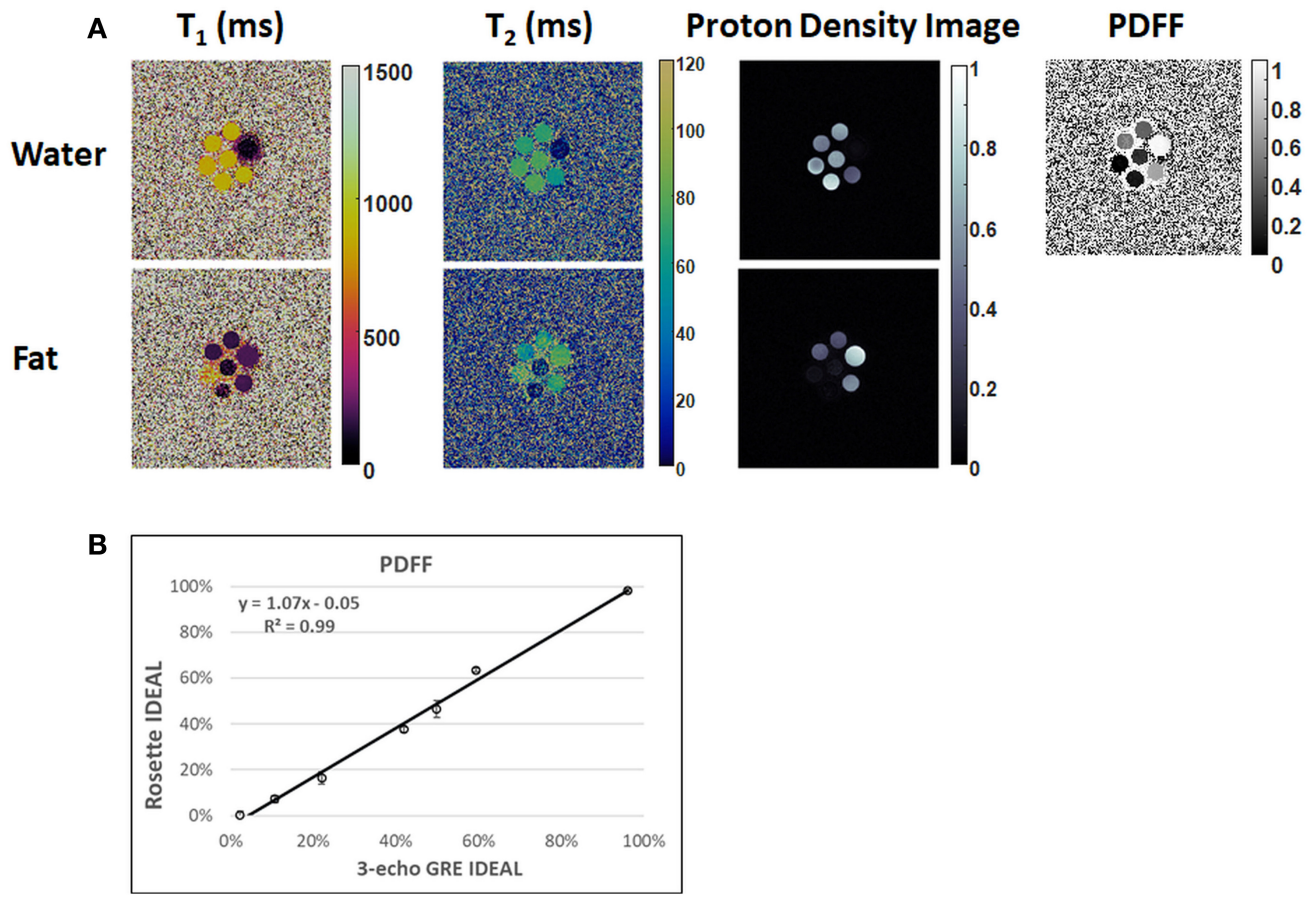
Results in the fat fraction phantom. **(A)** Water and fat specific T_1_ and T_2_ maps, proton density images, and the PDFF map generated by Hierarchical IDEAL using the rosette cMRF data. **(B)** PDFF measurements using rosette cMRF compared with reference values.

In the ISMRM/NIST system phantom, T_1_ and T_2_ measurements using rosette cMRF are in excellent agreement with the reference values (Supplementary Figure 1) (slope of best-fit line 1.02/1.01 for T_1_/T_2_, R^2^>0.99). In the fat fraction phantom, water and fat specific T_1_ and T_2_ maps, proton density images, and the PDFF map generated by Hierarchical IDEAL using the rosette cMRF data are shown in Figure 2. PDFF measurements using rosette cMRF agree well with 3-point GRE measurements (Figure 2B) (slope of best-fit line 1.07, R^2^ > 0.99). The water and fat specific T_1_ and T_2_ measurements in the fat fraction phantom are shown in Supplementary Figure 2. T_1_ and T_2_ measurements are consistent across the vials regardless of PDFF values (except in the high/low PDFF vials which have too little signal for either water or fat).

### Healthy subjects

**Figure 3:**
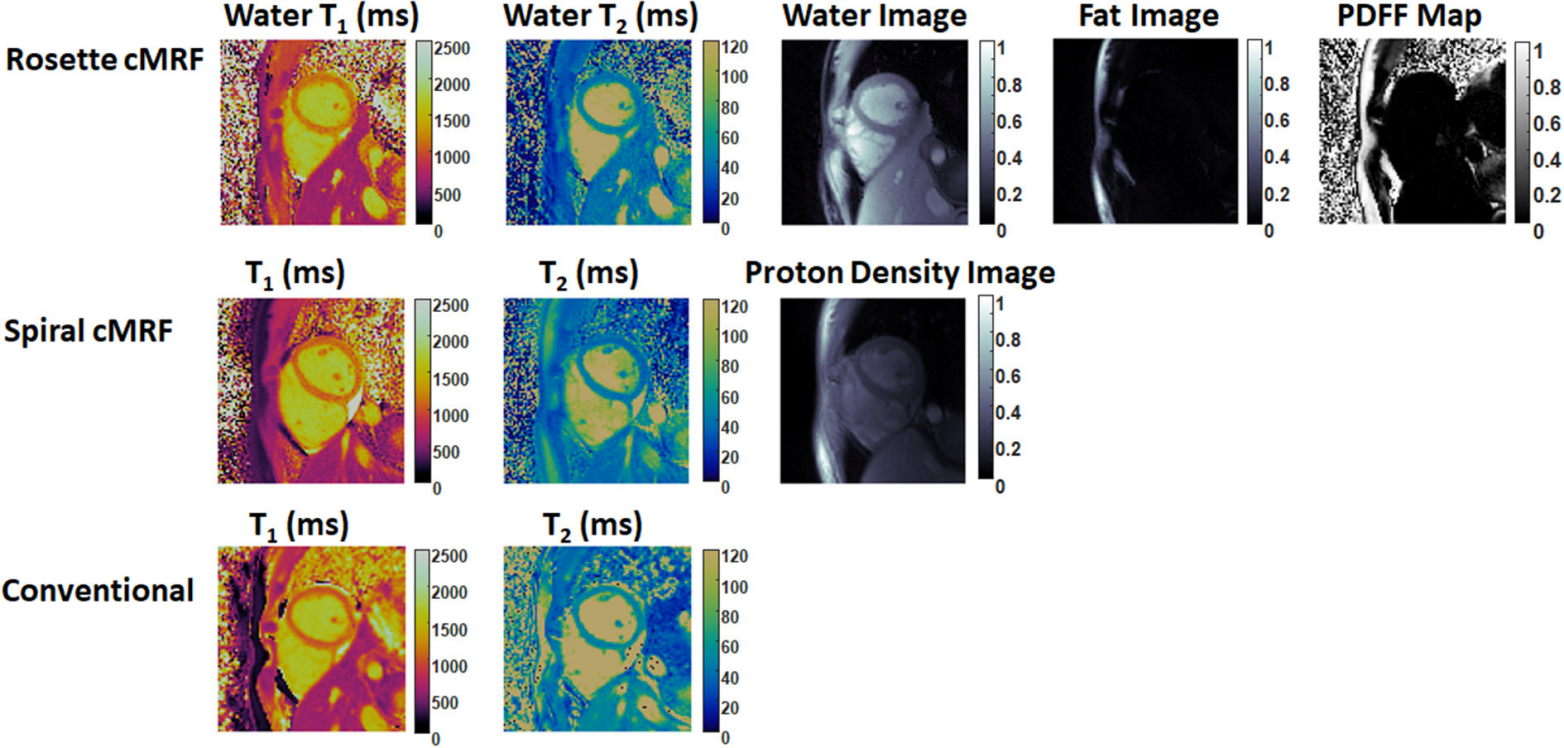
Representative T_1_ map, T_2_ map, water image, fat image, and PDFF maps in a healthy subject. T_1_ and T_2_ maps measured by spiral cMRF and conventional methods are shown for comparison. The field-of-view has been cropped to 150 × 150 mm^2^ to better visualize the heart.

**Figure 4:**
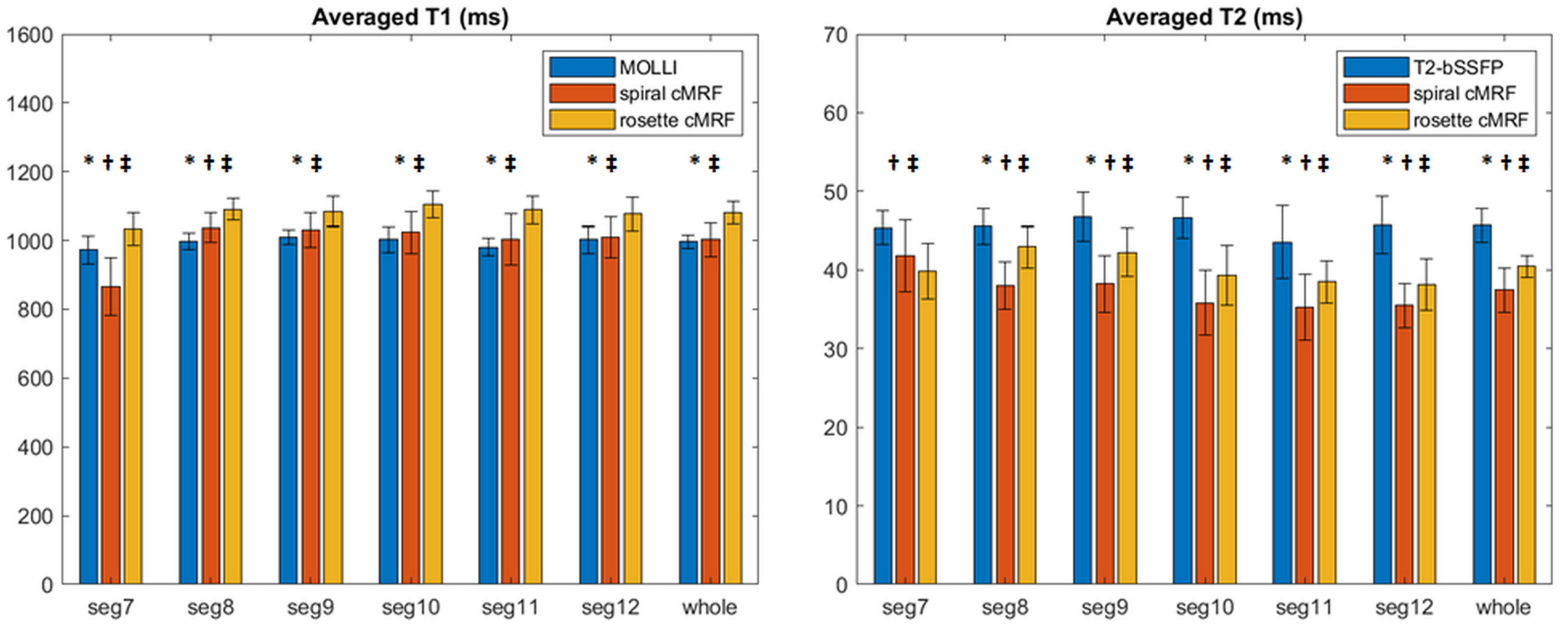
T_1_ and T_2_ values in 16 healthy subjects measured using conventional methods, spiral cMRF, and rosette cMRF. Measurements in segment 7–12 as well as over the entire myocardium are shown. *Significant difference between spiral and rosette cMRF. ^†^Significant difference between conventional method and spiral cMRF. ^‡^Significant difference between conventional method and rosette cMRF.

**Figure 5:**
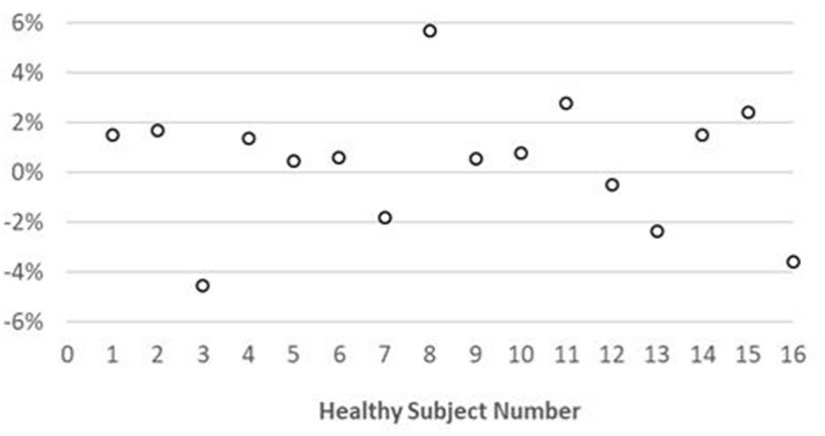
Individual PDFF over the myocardium measured using rosette cMRF in 16 healthy subjects.

Representative maps and images from one healthy subject are shown in Figure 3. The averaged T_1_ and T_2_ values of all subjects in each segment as well as in the entire myocardium are shown in Figure 4. Over the entire myocardium, spiral cMRF yielded similar T_1_ values (1,002 ± 50.6 ms) compared with MOLLI (996.5 ± 20.1 ms) while rosette cMRF generated significantly higher T_1_ values (1,081.1 ± 31.8 ms). Both spiral and rosette cMRF yielded significantly lower T_2_ values (spiral 37.4 ± 2.8 ms; rosette 40.5 ± 1.4 ms) compared with the conventional method (45.7 ± 2.2 ms) over the entire myocardium., and rosette cMRF generated significantly higher T_2_ values than spiral cMRF. Averaged PDFF over the myocardium was 0.4% among all subjects (ranging from −4.5% to 5.7%). Individual PDFF of the sixteen healthy subjects are shown in Figure 5.

In most of the individual segments (AHA segments 8-12), T_1_ and T_2_ values measured by the three methods show similar trends as in the entire slice. Spiral cMRF generated similar T_1_ values compared with MOLLI except for segment 7 and 8; rosette cMRF generated significantly higher T_1_ values compared with both MOLLI and spiral cMRF throughout all segments. Both spiral and rosette cMRF yielded significantly lower T_2_ values compared with conventional method throughout all segments. Rosette cMRF generated significantly higher T_2_ values than spiral cMRF in all segments except for segment 7. A cyclic pattern was noted in T_1_ and T_2_ measurements across the segments using all three methods, with lateral T_1_ and T_2_ slightly lower than septal ones. Variations in T_1_ and T_2_ across the segments are most pronounced in spiral cMRF (T_1_ ~170 ms; T_2_ ~6.6 ms), but smaller in rosette cMRF (T_1_ ~70 ms; T_2_ ~4.4 ms) and conventional methods (T_1_ ~38 ms; T_2_ ~3.2 ms).

With fat signals added back, the averaged rosette cMRF T_1_ measurements in segment 7 in all healthy subjects decreased from 1033.6 ± 48.4 ms to 1016 ± 85 ms; averaged T_2_ increased from 39.8 ± 3.6 ms to 43.5 ± 4.6 ms. A comparison of T_1_ and T_2_ measurements in all segments as well as the entire slice between spiral cMRF, rosette cMRF (with fat suppression), and rosette cMRF with fat signals added back is shown in Supplementary Figure 3.

### cMRF maps in patients

**Figure 6:**
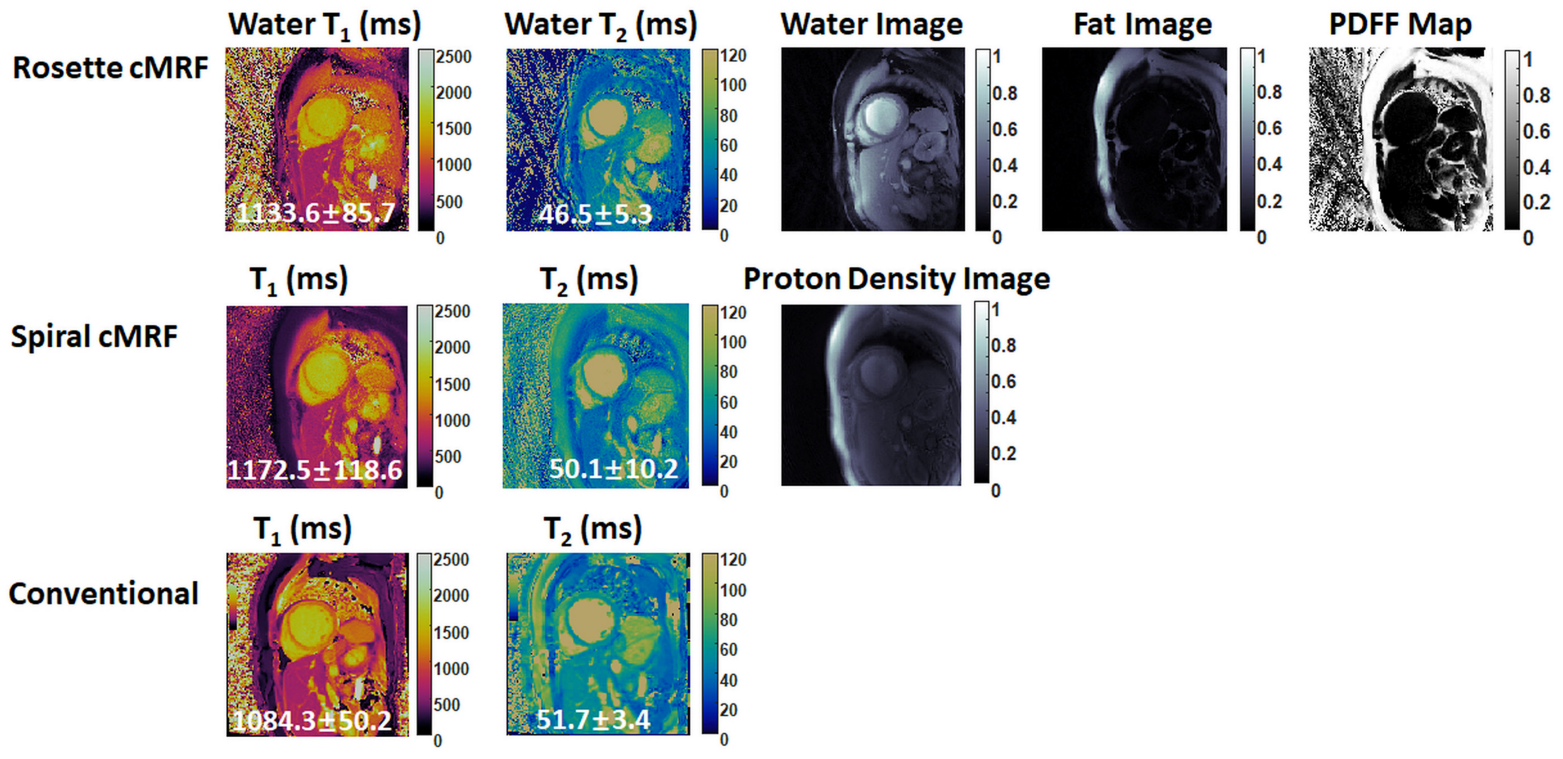
Pre-contrast results in the first cardiomyopathy patient acquired using rosette cMRF, spiral cMRF, and conventional methods. T_1_ and T_2_ values over the entire myocardium are shown in the maps.

**Figure 7:**
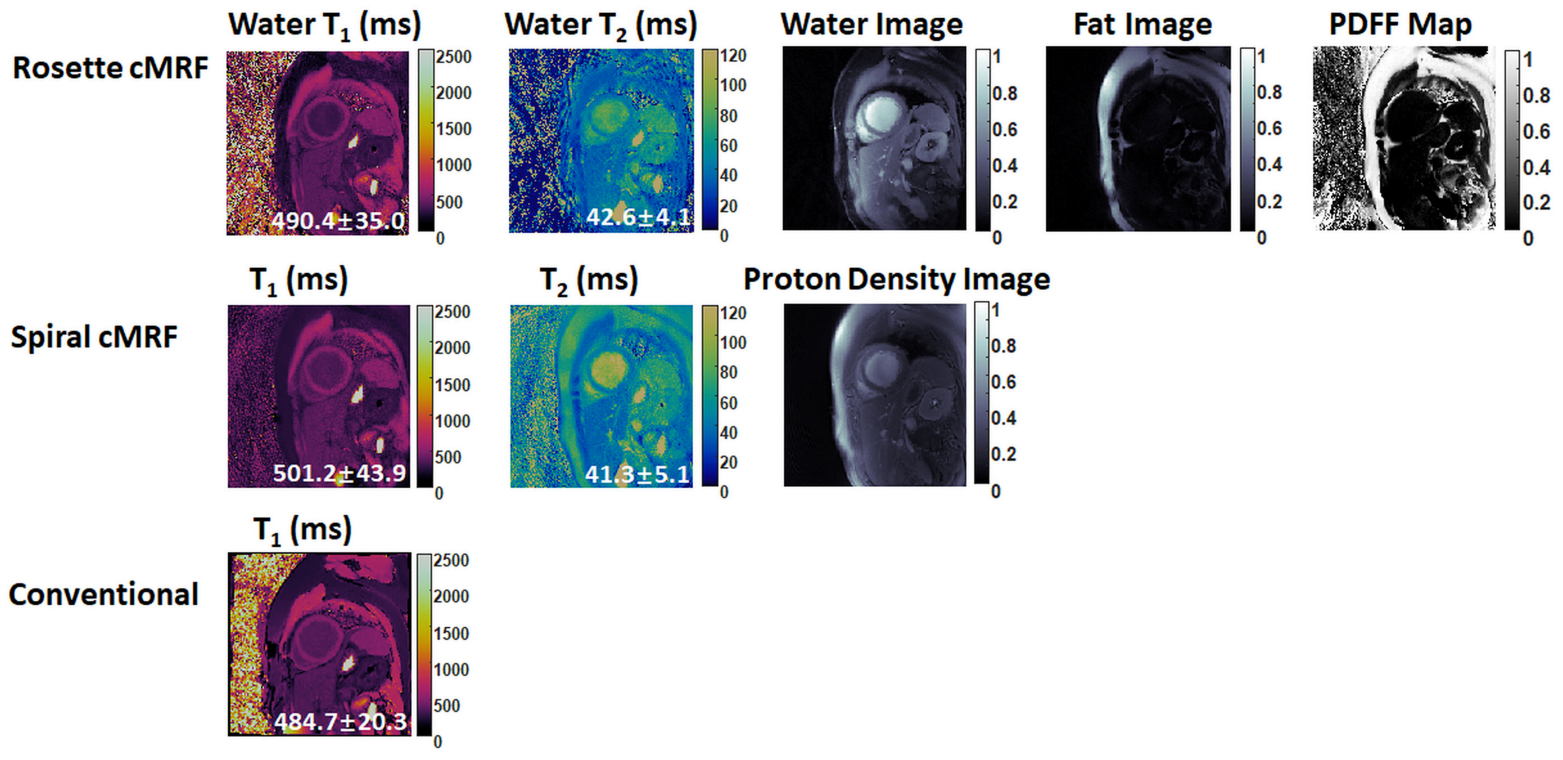
Post-contrast results in the first cardiomyopathy patient acquired using rosette cMRF, spiral cMRF, and conventional methods. T_1_ and T_2_ values over the entire myocardium are shown in the maps.

**Figure 8:**
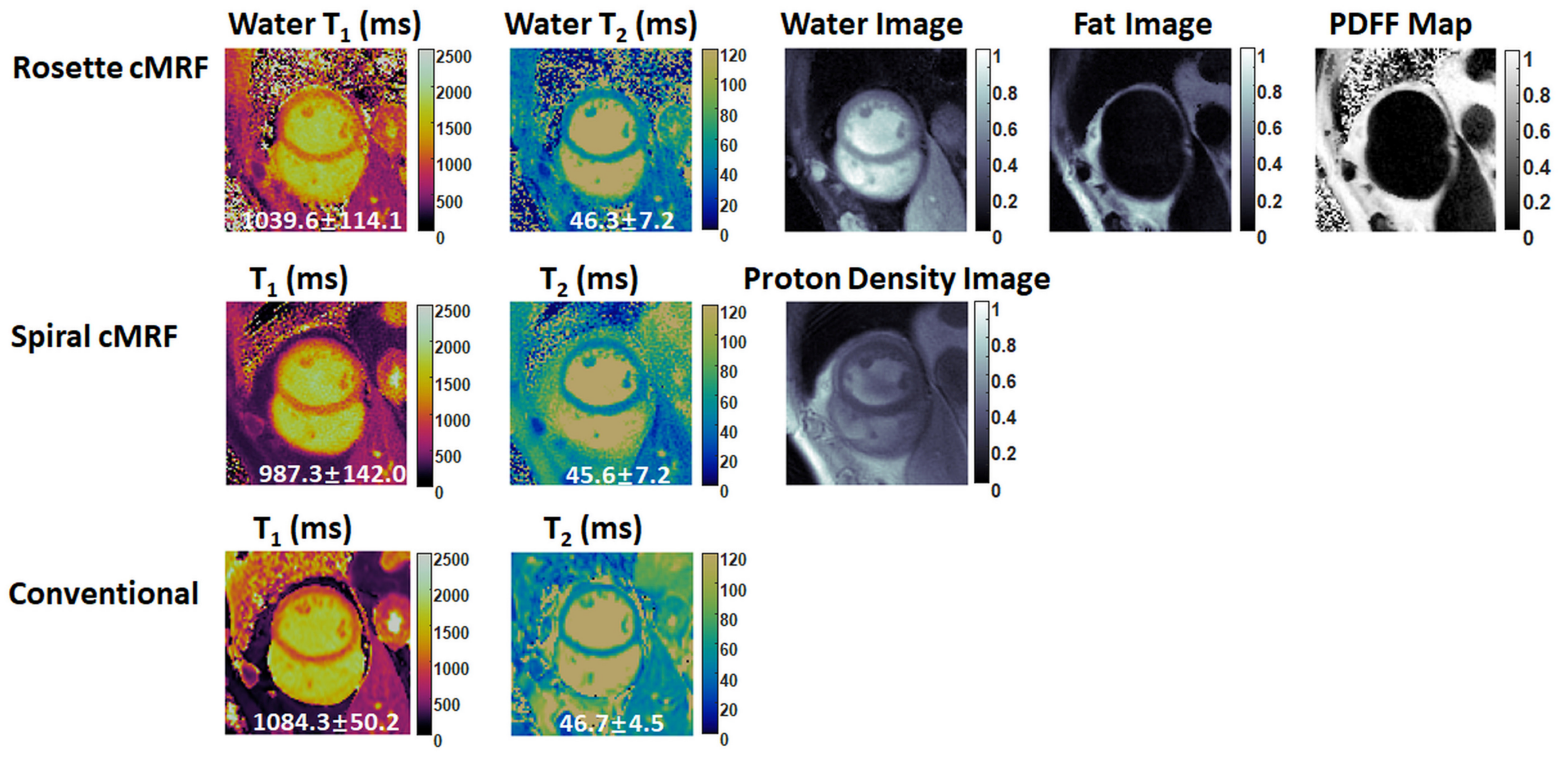
Pre-contrast results in the second cardiomyopathy patient acquired using rosette cMRF, spiral cMRF, and conventional methods. T_1_ and T_2_ values over the entire myocardium are shown in the maps. The field-of-view has been cropped to 150 × 150 mm^2^ to better visualize the heart.

**Figure 9:**
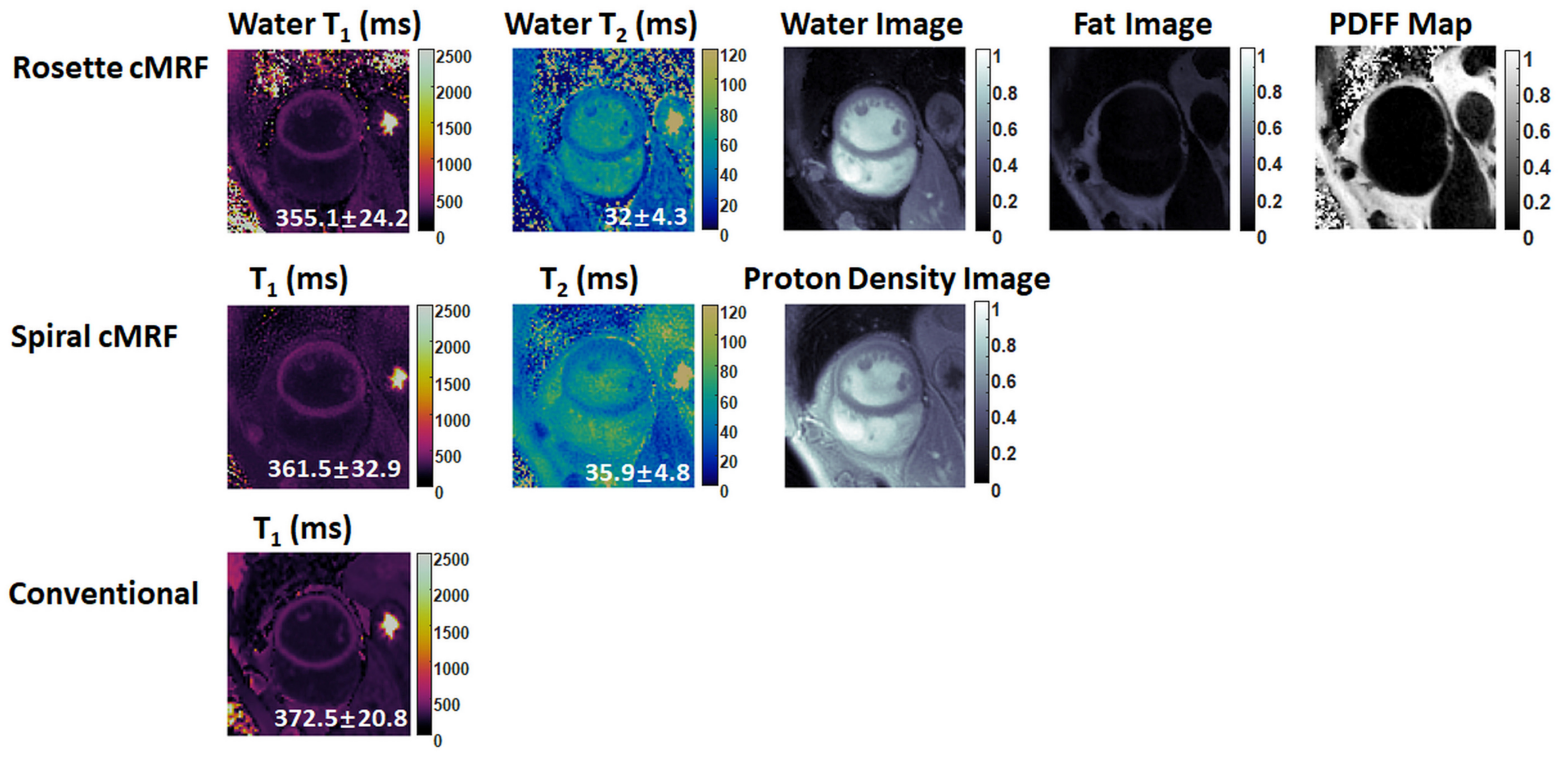
Post-contrast results in the second cardiomyopathy patient acquired using rosette cMRF, spiral cMRF, and conventional methods. T_1_ and T_2_ values over the entire myocardium are shown in the maps. The field-of-view has been cropped to 150 × 150 mm^2^ to better visualize the heart.

Figures 6, 7 show the pre- and post-contrast maps and images from one patient with cardiomyopathy, respectively. Elevated native T_1_ and T_2_ were observed using all three methods. PDFF over the myocardium measured by rosette cMRF pre- and post-contrast are 2.7 and 1.3%, respectively. Pre- and post-contrast results for the second patient are shown in Figures 8, 9. Myocardial PDFF measured by rosette cMRF pre- and post-contrast are 4.2 and 2.9%, respectively. Spiral cMRF maps exhibit blurring, especially in the T_2_ maps, caused by epicardial fat; rosette cMRF was able to achieve much clearer boundaries of the myocardium due to fat signal suppression.

## Discussion

The current study is an extension of previous work which used rosette cMRF for water-fat separation in addition to T_1_ and T_2_ mapping. The rosette trajectory originally designed for water-fat separation at 1.5T was optimized to improve off-resonance fat signal suppression from 86.5 to 94.7%. Note that the rosette trajectory was redesigned for PDFF measurement as compared to the trajectory used in (9), and thus all phantom and *in vivo* results presented here have no overlap with those reported in the previous work. A segmentation strategy was used to generate nine single echo images from each rosette readout, which could be used in conjunction with the Hierarchical IDEAL algorithm to enable PDFF quantification with no penalty in acquisition time. The first SVD coefficient images were used as the inputs to IDEAL in this work. Even though additional T_1_ and/or T_2_ corrections to these images were not found necessary for the specific sequence used in this study, potential T_1_ and T_2_ weighting of these images depending on the specific sequence structure (e.g. flip angles, magnetization preparation modules) might cause inaccuracy in PDFF quantification especially for low or high T_1_ (or T_2_) values. In contrast, the previous work was able to generate qualitative water and fat images but not quantitative PDFF maps; while the attempt was made to calculate PDFF maps from the water and fat proton density images generated by pattern matching, these values were found to be inaccurate, as these proton density images derived from the scaling factors between the signal time courses and the dictionary entries are not quantitative maps of proton density. In this work, a similar calculation relying on proton density images for PDFF using the newly designed rosette trajectory again resulted in inaccurate values (Supplementary Figure 4).

Other studies have explored water-fat separation and PDFF quantification using the MRF framework in static organs (24–28) and in the heart. For example, Dixon-cMRF using multi-echo radial readout has been proposed to quantify T_1_, T_2_, and PDFF simultaneously in the heart (8, 29). Compared to rosette cMRF, Dixon-cMRF generated comparable myocardial T_1_ (1032 ms) and T_2_ (42.1 ms) in healthy subjects in a similar acquisition time (~15 s) with slightly larger voxel size (2 × 2 × 8 mm^3^ in healthy subjects and 1.8 × 1.8 × 8 mm^3^ in patients). While Dixon-cMRF employed a different water-fat separation algorithm (graph cut) for PDFF quantification compared to the current study that used Hierarchical IDEAL, similar PDFF values were observed in healthy subjects (1.3% in the septum). Negative PDFF values were observed in the myocardium (Figure 5) due to the noise correction method performed in this study (20). When used in tissues with no fat content, this correction results in mean PDFF values of zero (with both positive and negative values possible). Unlike other MRF studies including Dixon-cMRF, advanced reconstruction methods such as low-rank reconstruction were not used in the current study to avoid long computation times with B_0_ correction. In this work, direct pattern match with SVD along the time dimension in conjunction with rosette MRF yields good image and map quality without the need for advanced reconstruction techniques. Incorporating low-rank reconstruction yields slightly smaller standard deviations in the T_1_ and T_2_ measurements with almost identical mean values and image quality at a price of much longer computing time (data not shown here).

T_1_ and T_2_ values measured by spiral cMRF and conventional methods in a large cohort of healthy subjects (*n* = 58) at 1.5T have been reported previously (30). Over the entire mid-ventricular slice, the current study found T_1_ values very close to the previous report and T_2_ values slightly lower in both spiral cMRF and conventional measurements. The trend that spiral cMRF with confounding factor corrections yielded higher T_1_ values than MOLLI and lower T_2_ values than T_2_-prep bSSFP method is also consistent with previous reports (16, 30). Similar to the previous rosette cMRF work (9), the current study found that rosette cMRF yielded ~3 ms higher T_2_ values than spiral cMRF over the entire myocardium. However, the significant difference between rosette and spiral cMRF T_1_ measurements observed in the current study was not found previously, possibly due to a much smaller number of subjects in the previous work (9).

While the previous rosette cMRF work only reported T_1_ and T_2_ values over the entire myocardium, the current study also examined each AHA segment of the mid-ventricular slice. Interestingly, segment 7 shows more pronounced difference in T_1_ measurements and an opposite trend in T_2_ measurements compared to the other segments regarding the comparison between spiral and rosette cMRF. Given that segment 7 (anterior wall) is surrounded by more epicardial fat than the other segments in the healthy subjects, and fat has T_1_ of 300~370 ms (lower than myocardium) and T_2_ of ~53 ms (higher than myocardium) at 1.5T (31), the higher T_1_ and lower T_2_ measured by rosette cMRF are possibly due to reduced fat contamination and may potentially be more accurate compared to spiral cMRF measurements. This hypothesis was also verified by the fact that T_1_ in segment 7 was decreased and T_2_ was increased when fat signals were added back to rosette cMRF data retrospectively (Supplementary Figure 3). The fact that rosette cMRF yielded smaller variations in T_1_ and T_2_ across cardiac segments compared to spiral cMRF (Figure 4) could also be evidence of effective fat signal suppression and more reliable T_1_ and T_2_ mapping. Note that difference between spiral and rosette cMRF measurements was still observed after fat signals were added back to the rosette data, indicating fat is not the only factor causing the difference. B_0_ field inhomogeneity, which was not modeled in this simulation, might play a role because it causes blurring in spiral images but signal loss in rosette images. Spiral and rosette trajectories may also react to flow differently due to their different gradient waveforms and gradient moments, resulting in variations in T_1_ and T_2_ measurements.

Preliminary results from cardiomyopathy patients are shown in this study. Both spiral and rosette cMRF were able to detect abnormal T_1_ and T_2_ values, while rosette cMRF potentially provided better image quality by suppressing fat signals in the water T_1_ and T_2_ maps. Studies with a larger cohort of cardiac patients are on-going to validate the proposed method in a variety of cardiac diseases.

In addition to T_1_ and T_2_, ${\text{T}}_{2}^{*}$ is also an important tissue property reflecting iron load in the myocardium (32). Given the multi-echo acquisition nature of rosette trajectories, ${\text{T}}_{2}^{*}$ quantification in the heart and liver has been shown feasible using rosette trajectories (33). Even though the current study did not aim at ${\text{T}}_{2}^{*}$ quantification and thus used a relatively short rosette readout, future work will explore the quantification of T_1_, T_2_, ${\text{T}}_{2}^{*}$ and PDFF simultaneously using either a long rosette readout (34) or multi-echo radial readout (35) in the MRF framework.

There are a few limitations of the current study. First, even though the accuracy of PDFF quantification was validated in fat fraction phantoms, *in vivo* validation of PDFF measurements was not performed due to unavailability of the clinical PDFF mapping sequences. Second, repeatability of rosette cMRF was not tested in healthy subjects. Third, the image quality in patient data was not assessed by cardiologists using a systematic approach such as a Likert scale. Future studies will aim to address these aspects.

## Conclusion

In conclusion, rosette cMRF is a promising method for efficient cardiac tissue characterization through the simultaneous quantification of myocardial T_1_, T_2_, and PDFF.

## Data availability statement

The original contributions presented in the study are included in the article/Supplementary material, further inquiries can be directed to the corresponding author.

## Ethics statement

The studies involving human participants were reviewed and approved by Institutional Review Boards of the University of Michigan Medical Campus. The patients/participants provided their written informed consent to participate in this study.

## Author contributions

YL and JH contributed to pulse sequence development and data acquisition. YL performed data reconstruction, statistical analysis, wrote the draft of the manuscript, and created figures. All authors contributed to conception of the study, manuscript revision, and approved the submitted version.

## Funding

JH is supported by NIH/NHLBI R01HL163030. This work was supported by NSF/CBET 1553441, NIH/NHLBI R01HL094557, and Siemens Healthineers (Erlangen, Germany). Siemens Healthineers was not involved in the study design, collection, analysis, interpretation of data, the writing of this article or the decision to submit it for publication.

## Conflict of interest

The authors declare that the research was conducted in the absence of any commercial or financial relationships that could be construed as a potential conflict of interest.

## Publisher's note

All claims expressed in this article are solely those of the authors and do not necessarily represent those of their affiliated organizations, or those of the publisher, the editors and the reviewers. Any product that may be evaluated in this article, or claim that may be made by its manufacturer, is not guaranteed or endorsed by the publisher.
