# Supplementary material for: Cardiac MRF using rosette trajectories for simultaneous myocardial T1, T2, and proton density fat fraction mapping
Source: Front Cardiovasc Med. 2022 Sep 20;9:977603. doi: 10.3389/fcvm.2022.977603 (PMC9530568; doi:10.3389/fcvm.2022.977603)
Supplement: Supplementary file 1 [file Table_1.DOCX]

Supplementary Material


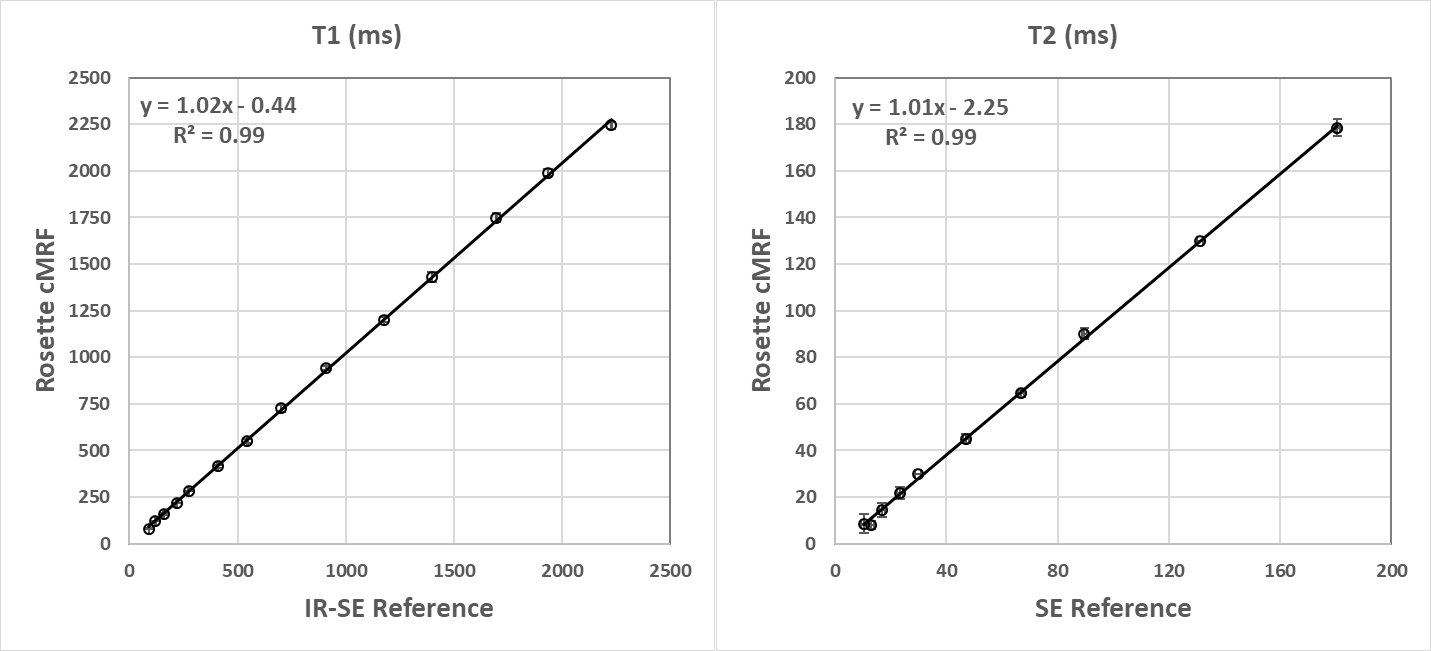


**Supplementary Figure 1.** T_1_ and T_2_ measurements in the T_2_ layer of the ISMRM/NIST system phantom using rosette cMRF compared with reference values acquired using inversion recovery spin echo and single echo spin echo methods.


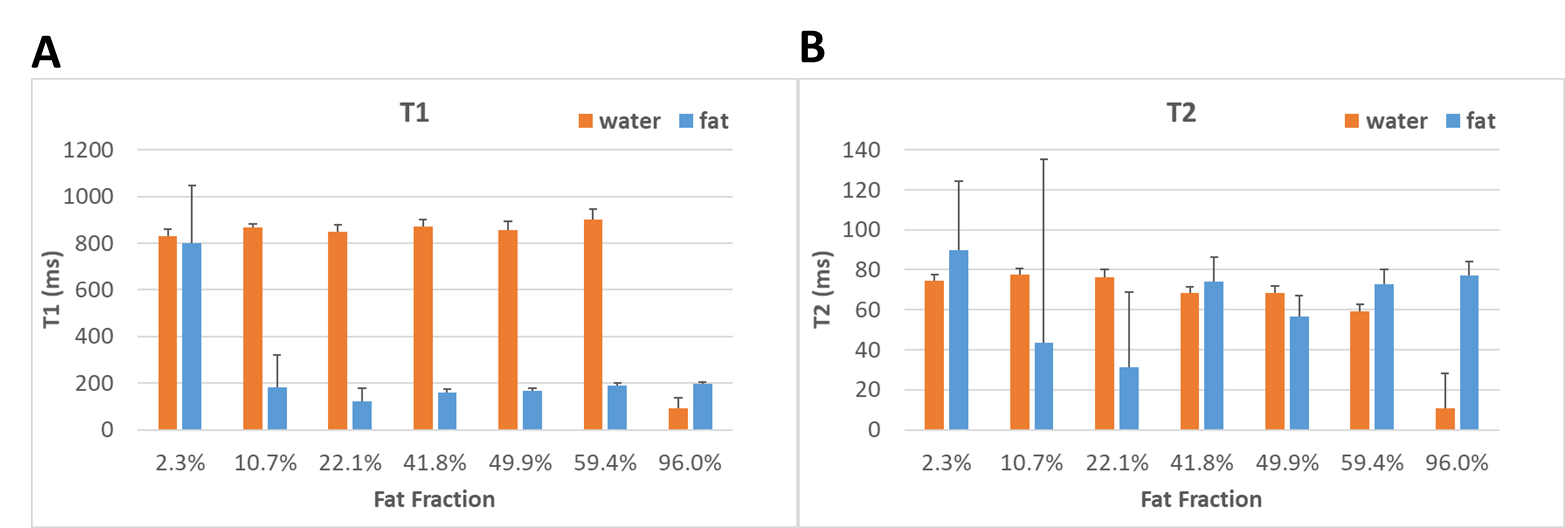


**Supplementary Figure 2.** Water and fat specific T_1_ (A) and T_2_ (B) measurements in the fat fraction phantom using rosette cMRF. Note that water/fat T1 and T2 measurements in the high/low PDFF vials are not accurate due to signal suppression.


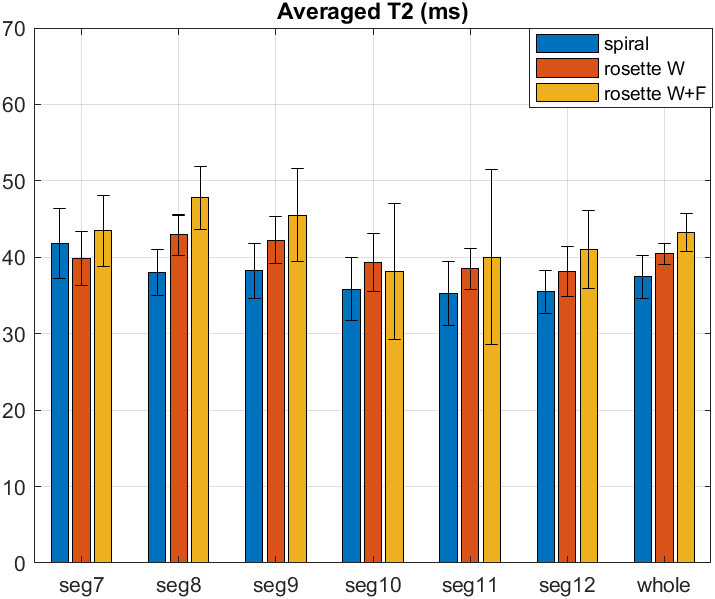

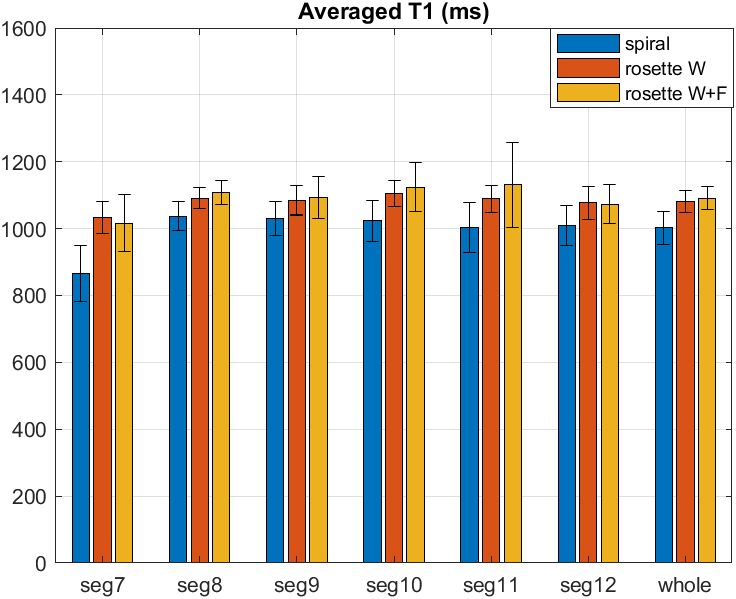


**Supplementary Figure 3.** T_1_ and T_2_ values in 16 healthy subjects measured using spiral cMRF, rosette cMRF with fat suppression, and rosette cMRF with fat signals added back retrospectively. Measurements in segment 7 to 12 as well as over the entire myocardium are shown.


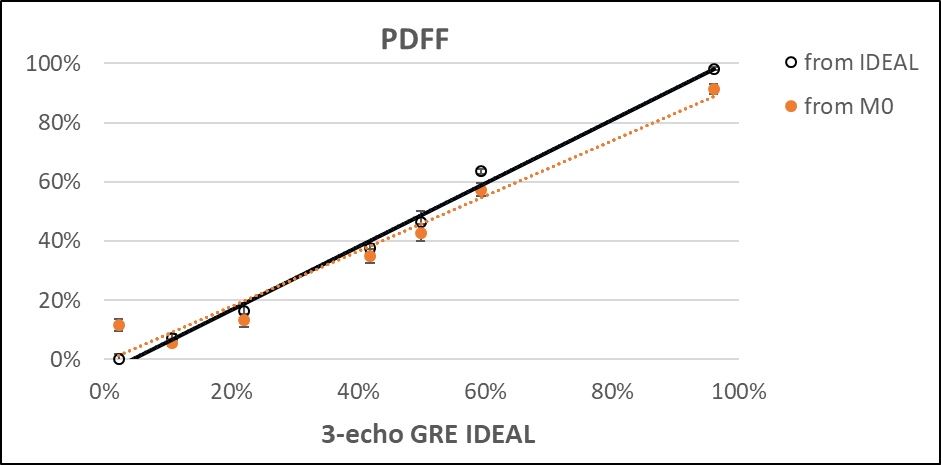


**Supplementary Figure 4.** A comparison between the PDFF values calculated using IDEAL (black dot) and using proton density images from MRF pattern matching (orange dot) against the reference values in the fat fraction phantom.
